# A Pleomorphic Puzzle: Heterogeneous Pulmonary Vascular Occlusions in Patients with COVID-19

**DOI:** 10.3390/ijms232315126

**Published:** 2022-12-01

**Authors:** Jeeshan Singh, Irmgard Herrmann, Aparna Mahajan, Christine Schauer, Xiaomei Shan, Arndt Hartmann, Ralf J. Rieker, Katja Evert, Christina Falkeis, Elisabeth Naschberger, Saskia von Stillfried, Peter Boor, Luis E. Muñoz, Georg Schett, Martin Herrmann, Jasmin Knopf

**Affiliations:** 1 Department of Internal Medicine 3—Rheumatology and Immunology, Friedrich-Alexander University Erlangen-Nürnberg, and Universitätsklinikum Erlangen, 91054 Erlangen, Germany; 2 Deutsches Zentrum für Immuntherapie (DZI), Friedrich-Alexander University Erlangen-Nürnberg and Universitätsklinikum Erlangen, 91054 Erlangen, Germany; 3 Institut für Pathologie, Universitätsklinikum Erlangen, Friedrich-Alexander-Universität Erlangen-Nürnberg, 91054 Erlangen, Germany; 4 Institut für Pathologie, Universität Regensburg, 93053 Regensburg, Germany; 5 Department of Pathology, Klinikum Bayreuth, 95445 Bayreuth, Germany; 6 Division of Molecular and Experimental Surgery, Translational Research Center, Department of Surgery, University Medical Center Erlangen, Friedrich-Alexander-Universität Erlangen-Nürnberg, 91054 Erlangen, Germany; 7 Institute of Pathology, University clinic of the RWTH Aachen, 52074 Aachen, Germany

**Keywords:** SARS-CoV-2, COVID-19, immunothrombosis, occlusions, pleomorphism, native endogenous fluorescence, age, gender

## Abstract

Vascular occlusions in patients with coronavirus diseases 2019 (COVID-19) have been frequently reported in severe outcomes mainly due to a dysregulation of neutrophils mediating neutrophil extracellular trap (NET) formation. Lung specimens from patients with COVID-19 have previously shown a dynamic morphology, categorized into three types of pleomorphic occurrence based on histological findings in this study. These vascular occlusions in lung specimens were also detected using native endogenous fluorescence or NEF in a label-free method. The three types of vascular occlusions exhibit morphology of DNA rich neutrophil elastase (NE) poor (type I), NE rich DNA poor (type II), and DNA and NE rich (type III) cohort of eleven patients with six males and five females. Age and gender have been presented in this study as influencing variables linking the occurrence of several occlusions with pleomorphic contents within a patient specimen and amongst them. This study reports the categorization of pleomorphic occlusions in patients with COVID-19 and the detection of these occlusions in a label-free method utilizing NEF.

## 1. Introduction

More than 6.43 million people have died (as of August 2022) since the first reports of severe acute respiratory syndrome coronavirus-2 (SARS-CoV-2) [1]. Although fever, sore throat, dry cough, fatigue [2], anosmia [3] myalgia [4], diarrhea, and anorexia [5] remain the most commonly reported mild symptoms of the original strain of coronavirus disease 2019 (COVID-19) first emerging in Wuhan, the severe course of the disease in critical patients is often associated with thrombosis, with thrombotic complications occurring in up to 1 in 3 patients [6,7].

The binding of SARS-CoV-2 to type II pneumocytes in alveoli triggers excessive migration of innate immune system cells, mainly monocytes, macrophages, and neutrophils, which are the chief components in the immunologically mediated thrombi formation via multifactorial processes. Current research proposes various mechanisms that contribute to the formation of immunothrombosis, e.g., (I) the release of pro-inflammatory cytokines, (II) the activation of complement, and (III) the formation of neutrophil extracellular traps (NETs) [8]. While several pathological mechanisms are responsible for immunothrombosis in patients with COVID-19, NETs are composed of decondensed chromatin associated with histones and granular proteins such as neutrophil elastase (NE). Visualization techniques used for detecting and quantifying NETs are usually based on the detection of citrullinated histone H3 (citH3) bound to DNA and not NE. It is well described in the literature that neutrophils via NET formation play a cornerstone role in immunothrombosis. NE is a key enzyme of neutrophils that is predominantly used as a neutrophil detection marker, but unfortunately NETs positive for NE are poor quality markers of neutrophil-induced immunothrombosis.

Neutrophils are one of the most crucial and abundant leukocyte populations in humans, which perform critical functions such as restricting the spread of invading pathogens and resolving inflammation. However, the pathological nature of NETs has been recognized in orchestrating the occurrence of several disorders [9].

The exuberant aggregation of NETs (aggNETs) has been shown to obstruct the terminal ducts of meibomian glands resulting in meibomian gland dysfunction (MGD) and causing ocular surface inflammation [10]. In addition, NETs foster the formation of gallstones by aiding the accumulation of calcium crystals and biliary cholesterol [11]. Furthermore, exaggerated NETs cause epithelial cytotoxicity and release proteases, injuring the basal lamina [12,13,14]. This pathological feature of occlusive NETs was also reported to congest the vasculature during the first wave of the pandemic caused by the original SARS-CoV-2 strain [15,16,17].

Infection by SARS-CoV-2 of alveolar endothelia causes the neutrophils to arrive at the site of injury. As a result, NETs are quickly formed to eliminate and prevent the dissemination of SARS-CoV-2 and promote vascular occlusion. NET-mediated occlusions result from the complex intertwined role of platelet binding and aggregation, or the interactions with the von Willebrand Factor (vWF) factor, released from the endothelia and platelets. This interaction causes the further attachment of platelets and the formation of fibrin. Neutrophil upregulation of tissue factor (TF), and subsequent NET formation decorated with TF play a critical role in the thrombi formation. The serine protease NE released during NET formation inhibits anticoagulation by degrading thrombomodulin and tissue factor pathway inhibitor (TFPI) [8]. NETs can also activate both intrinsic and extrinsic coagulation cascades through the release of microparticles enclosing tissue factor (TF) by neutrophils, coagulation factor FXII activation, and fibrinolysis inhibition promoting immunothrombosis [18].

On the other hand, the activation of coagulation cascades after the infection of endothelial cells with SARS-CoV-2 also entails an interplay of platelet binding, fibrin formation, and erythrocytes clotting, generating a systemic occlusion that imparts severe effects [19]. Additionally, several studies have demonstrated microvascular thrombi in patients with COVID-19 harboring fibrin deposits, and citrullinated histone 3 (citH3) neutrophils mediating the formation of NETs. In addition, NETs co-localized with platelet-derived thrombotic factors and intravascular clots, resulting in extensive aggregation of neutrophils, have also been reported [15,20].

Autopsies and histological investigations have shown venous thromboembolism, arterial thrombosis, myocardial infarction, stroke, and macro- and microvascular occlusions as thrombotic manifestations of COVID-19 [21], and indicated unusual pathophysiology in patients with severe COVID-19. Lung specimens from autopsies of three patients with COVID-19 revealed pulmonary capillaries infiltrated with neutrophils, migration of neutrophils into the alveolar region, and neutrophilic mucositis [22]. Histological analysis of lung tissues from six patients who succumbed to COVID-19 also displayed neutrophilic pneumonia [23]. Post-mortem analysis of lung specimens from patients with COVID-19 exhibited neutrophils extruding NETs composing microvascular thrombi incorporating platelets, resulting in pulmonary dysfunction and death [20,24]. Furthermore, a pleomorphic morphology of NETs congesting the pulmonary vessels has been reported as a consequence of infection with the original strain of SARS-CoV-2 [15]. During former studies, we investigated 418 vessels of various sizes from autopsy-derived lung specimens from patients who succumbed to death due to the infection with the original strain of COVID-19 and compared these with 84 vessels from lung specimens of two non COVID-19 pathologies. We found that of the 418 pulmonary vessels analyzed in COVID-19 infected lungs and 84 vessels of controls, 328/418 (78%) and 19/84 (22%) of the vessels showed occlusions, respectively. For vessels below 40 µm diameter, the rate of occlusions was even higher 212/240 (88%) for patients with COVID-19 but similar for the controls 8/46 (17%), showing that small vessel occlusions were predominant in patients with the original strain of COVID-19 and with a unique dynamic morphology [15].

This histology-based study aims to characterize the previously reported pleomorphic occlusions in the lungs of patients with COVID-19 who succumbed in the first wave of the pandemic due to the circulation of the original strain of SARS-CoV-2. The striking heterogeneous appearance of vascular occlusions in these patients is determined by various levels of NETs-associated proteins, DNA, platelets, fibrinogen, and hemoglobin within the vasculature. Hemoglobin has previously served as a label-free marker for the detection of red blood cells due to its native endogenous fluorescence (NEF) properties [25,26]. Therefore, we could detect most of the vascular occlusions due to the NEF emerging from erythrocytes accumulated in the vasculature without any exogenous-fluorophore labeled antibody. We also report age and gender influencing the type of pleomorphic occlusions in patients with COVID-19.

## 2. Results

In order to characterize how NETs and proteins of the coagulation cascade provoke dynamic vascular occlusions, we stained lung tissue sections from patients with COVID-19 for neutrophil elastase (NE), platelets, and fibrinogen-α using fluorophore-labeled antibodies. Vascular occlusions were additionally detectable at excitation (ex). A measure of 488/emission (em) 525 nm without immunofluorescence staining suggests a population of cells that emits native endogenous fluorescence (NEF) at 488/525 nm.

### 2.1. NEF of Vascular Occlusion Shows Size Heterogeneity

**Figure 1:**
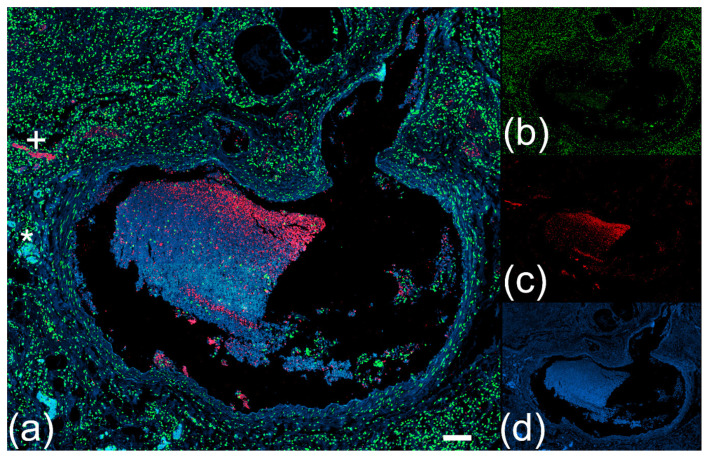
Native endogenous fluorescence (NEF) detects heterogeneous shapes of pulmonary vascular occlusions in paraffin lung specimens from patients with COVID-19. (**a**) Analyzed tissue section shown as a merged image of the three right displays shows a large vascular occlusion surrounded by several smaller vessels with circular and longitudinal vascular cross-sections marked as (*) or (+), respectively. The large occluded vessel is shown with propidium iodide for DNA (**b**, green), an antibody against neutrophil elastase (NE) (**c**, red), and NEF (**d**, blue). The scale bar represents 700 µm.

**Figure 2:**
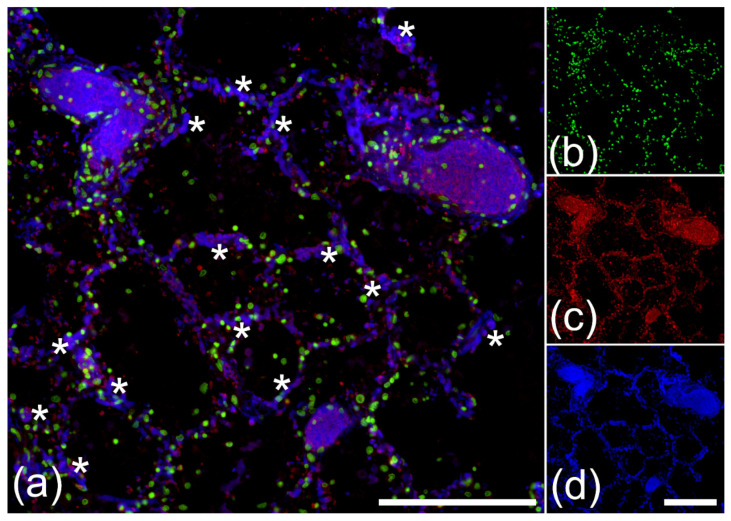
Microvascular occlusions in patients with COVID-19 identified by NEF in the alveolar region. (**a**) Analyzed tissue area is shown as a merged image of the three right displays exhibiting several vascular occlusions in the alveolar space, including microvascular structures. Several small vessels display a robust NEF signal marked with (*) and are positive for NE. These marked microvascular occlusions were stained for DNA with propidium iodide (**b**, green), NE (**c**, red), and NEF (**d**, blue). The scale bars represent 250 µm.

We utilized the properties of NEF to detect vascular occlusions in lung specimens from 17 patients with COVID-19. Occluded vessels were detected by their high NEF signal intensities showing widespread clots congesting a magnitude of circular (*) and longitudinal cross-sectional vessels (+) in the macro and microvasculature (Figure 1a and Figure 2a) of the different patients. In addition, NEF-detected vascular occlusions were analyzed for NE and extracellular chromatin indicative of NET formation. Immunofluorescence microscopy exhibited NEF-detected occlusions with an abundance of extracellular chromatin (Figure 1b and Figure 2b) decorated with high amounts of NE (Figure 1c and Figure 2c), highlighting the characteristic feature of NETs. Lung tissue area at 488/525 nm showed the vast presence of vascular occlusions in patient specimen (Figure 1d and Figure 2d).

**Figure 3:**
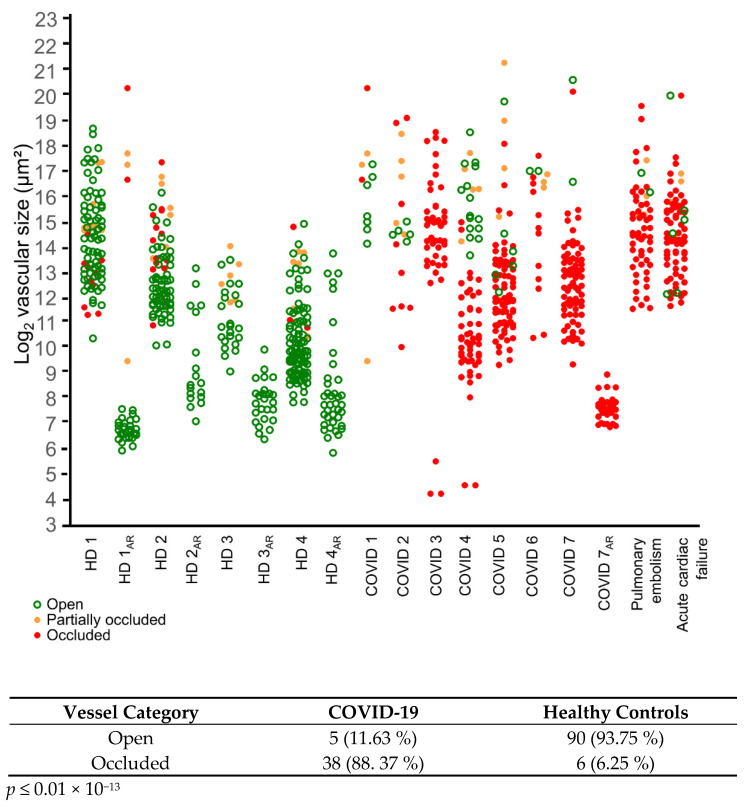
NEF detects vessels of heterogeneous areas in paraffin embedded lung specimens from patients with COVID-19 and non-COVID pathologies. (**Upper** panel) Analysis of various vessel areas using vessel morphometry shows open, partially occluded, and completely occluded vessels in green, orange, and red, respectively. Imaging with NEF detected these vessels in lung specimens from patients with COVID-19 (n = 7), pulmonary embolism (n = 1), and acute cardiac failure (n = 1). Compared to healthy control lung tissue sections or healthy donors (HD) (n = 4), NEF detected increased occluded vessels in patients with COVID-19. The morphometric analyses also showed that NEF-detected occluded vessels in the alveolar region (AR) of patients with COVID-19, showing this region to be mainly affected. (**Lower** panel) A tabular representation shows that NEF-detected occlusions in patients are higher in percentage than in healthy controls. The *p*-values were determined using Fisher’s exact test: *p* ≤ 0.01 × 10^−13^.

The detection of occluded vessels of heterogenous size using NEF is useful for morphometric analyses of tissue biopsies from patients with COVID-19 and can also be used for a general assessment of occluded vasculature as shown in Figure 3. Occluded vessels of various sizes were also detected in tissue biopsies from patients with pulmonary embolisms and acute cardiac failure, whereas the vessels of healthy donors were mostly open (Figure 3). NEF’s employability can also be extended to other pathologies, such as popliteal artery aneurysms (Figure S1). The reliable detection of vascular occlusions by NEF is confirmed by hematoxylin and eosin (H&E) staining (Figure S2a,b) of the consecutive area, as displayed in Figure 1 and Figure 2. In addition, we showed that NEF detects heterogeneity in vascular occlusions. A comparative analysis using H&E staining v/s NEF was made by identifying occluded to non-occluded vessels showing few intra-luminal erythrocytes in healthy lung tissue specimens (Figure S3–S5), confirming accurate detection by NEF of both occluded and non-occluded vessels.

### 2.2. NEF from Vascular Occlusions Is Associated with Hemoglobin-α

To investigate the origin of NEF in vessel occlusions of patients with COVID-19, lung specimens were stained for hemoglobin-α and imaged using immunofluorescence microscopy.

**Figure 4:**
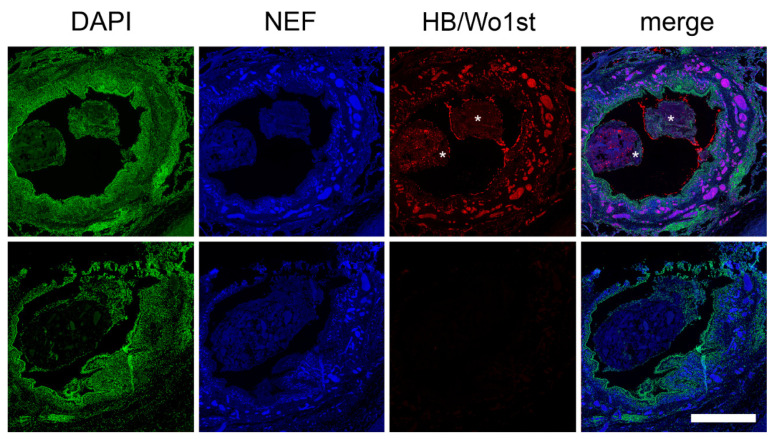
NEF signal from occluded vessels mainly originated due to erythrocyte hemoglobin-α (HB). Microphotographs of a large, partially occluded vessel surrounded by extensive microvessel occlusions show that the NEF signal (blue) concurs with hemoglobin-α (HB) positive clots (red) co-localized areas are shown in purple in the merged column. In contrast, some occlusions in the merged section show NEF emerging from regions not associated with HB (marked with asterisks). The scale bars represent 100 µm.

**Figure 5:**
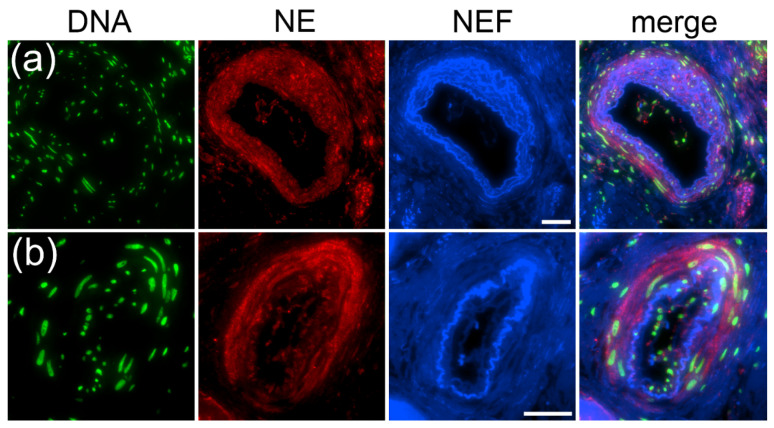
NEF-identified arterial vessel walls were neutrophil elastase (NE)-rich in biopsy samples from a patient with COVID-19. Two different blood vessels identified in patients with COVID-19 using NEF show arterial vessel walls are decorated with NE (**a**,**b**). Stains: DNA by propidium iodide (DNA, green), NE by fluorescence-labeled antibodies (red), and native endogenous fluorescence (NEF, blue). The merged fluorescence is displayed in the right column (merged). The scale bar represents 100 µm.

Surprisingly, few vascular occlusions emitting NEF at 488/525 nm in lung specimens were hemoglobin-α (HB) negative, marked with asterisks (Figure 4). However, the majority of the vessel occlusions showed co-localization for both NEF and HB, as observed in purple in the merged column of Figure 4. These findings suggest that hemoglobin from erythrocytes is primarily responsible for the emission of NEF in vascular occlusions. Furthermore, elastin and collagen from the blood vessels have been utilized as native endogenous fluorophores to differentiate between different blood vessels [20]. Using immunofluorescence microscopy, we also identified arterial vessels using NEF in a stained tissue for NE and DNA (Figure 5).

At 488/525 nm of wavelength, blood vessels emitting NEF-differentiated blood vessels and exhibited the typical curved, fibrous layers of elastin-composed thick arterial vessels rich in NE (Figure 5a,b). Interestingly, a broad view of this lung tissue area showed low or no presence of platelets and fibrinogen in the vessel wall (Figure S6).

### 2.3. NEF-Detected Vascular Occlusions Exhibit Pleomorphic Composition of Platelets and Fibrin

**Figure 6:**
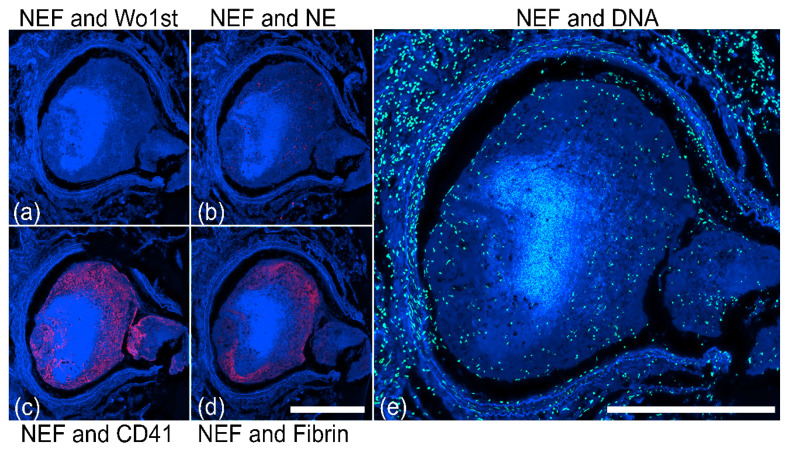
Immunofluorescence staining of a NEF-detected vascular occlusion in lung tissue section displaying blood coagulation factors and neutrophils. (**a**) NEF exhibited by an occlusion (blue) as a negative control for antibody staining. Immunofluorescence stainings shows an immunothrombosis for (**b**) NE deposits (red), (**c**) platelets (CD41, red), and (**d**) fibrin (red). (**e**) Immunothrombus shows nuclei of various cells stained by propidium iodide (green). The scale bars represent 2000 µm.

To characterize the dynamic feature of vessel occlusions in lung specimens from the same patient, we performed immunofluorescence staining using antibodies targeting CD41 and fibrinogen, both essential markers for blood coagulation, and NE for neutrophil detection. NEF-detected macrovascular occlusion (Figure 6) shows NE deposits scattered scarcely across the occlusion (Figure 6b), accompanied by layers of CD41 positive platelets (Figure 6c), and fibrinogen-α (Figure 6d) enclosing the core of the occlusion, and various other cell populations identified by the propidium-iodide DNA staining (Figure 6e)

**Figure 7:**
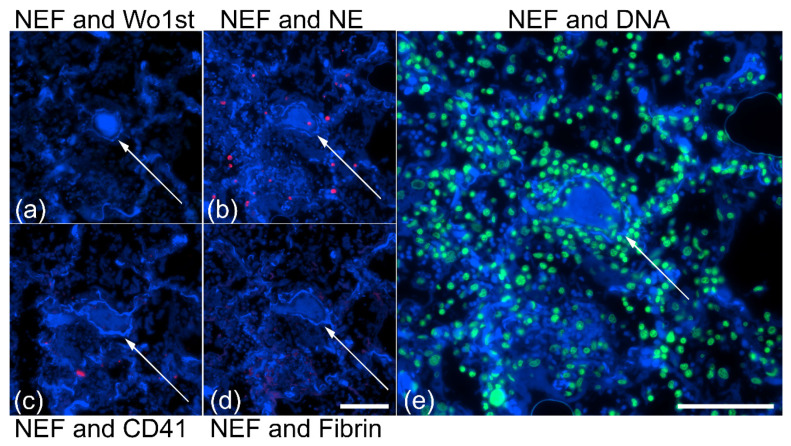
Stained NEF-detected microvascular occlusion in the alveolar region of patients with COVID-19 shows low NE expression and absence of platelets (CD41) and fibrin. (**a**) Microvascular occlusion (white arrow), detectable in NEF (blue) as a negative control for antibody staining. Microvessel occlusion stained shows (**b**) low NE expression (red) and the absence of both (**c**) platelets (red) and (**d**) fibrin (red). (**e**) Microvascular occlusion shows nuclei of various cells stained by propidium iodide (green). The scale bars represent 250 µm.

In contrast, occlusions in the microvasculature were morphologically different from macrovascular occlusion reported in Figure 6. The NEF-detected immunothrombosis showed few NE granules (Figure 7b) and the absence of platelets and fibrinogen (Figure 7c,d), but the presence of several cells shown by propidium-iodide DNA staining (Figure 7e), indicating heterogeneity in the composition of occlusions. The following sections will discuss a classification of different occlusion morphology or pleomorphism in more detail.

### 2.4. Type I Pleomorphism: DNA-Rich and NE-Poor

**Figure 8:**
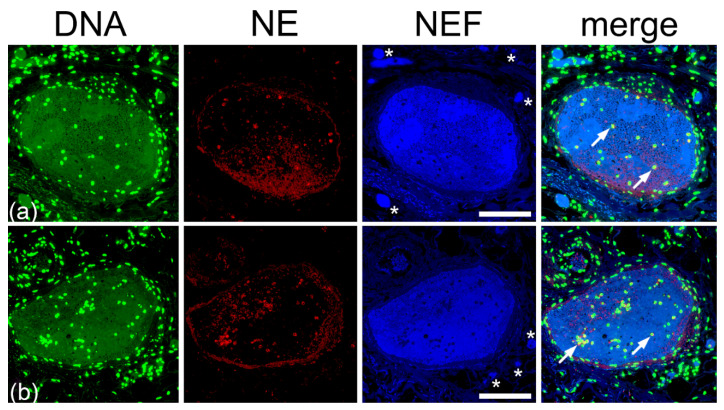
DNA-rich and NE-poor composition in vascular occlusion patients with COVID-19. (**a**,**b**) DNA-rich NE-poor occlusions show strong native endogenous fluorescence (NEF). The detected small vessels are marked with (*) asterisks in the column showing NEF. Arrows indicate intravascular neutrophils with intact nuclei in columns showing merged images. Stains: DNA by propidium iodide (DNA, green), NE by fluorescence-labeled antibodies (NE, red), and native endogenous fluorescence (NEF, blue). The right column displays merged fluorescence. The scale bars represent 100 µm.

NEF-detected vascular occlusions were analyzed for NE and DNA to comprehend occlusion pleomorphism in vessels of patients with COVID-19. Immunofluorescence staining in Figure 8 shows two clots in which propidium iodide-stained DNA of several cells are spread throughout and at the perimeter of the occlusion.

The DNA primarily co-localizes with NE, present in low amounts only at the margins of the occlusions and merely across the clot with a few NE-positive neutrophils (white arrows), suggesting a barrier composed of NETs, enclosing the occlusion and exhibiting a DNA-rich and NE-poor morphology (Figure 8a,b). The varying amounts of DNA and NE were the major components of the occlusions imparting pleomorphism to the occlusions and smaller amounts of platelets and fibrinogen were observed constituting the clots (Figure S7). The original morphology of the type I occlusions is shown using H&E staining in the supplementary material Figure S10a,b.

### 2.5. Type II Pleomorphism: NE-Rich and DNA-Poor

**Figure 9:**
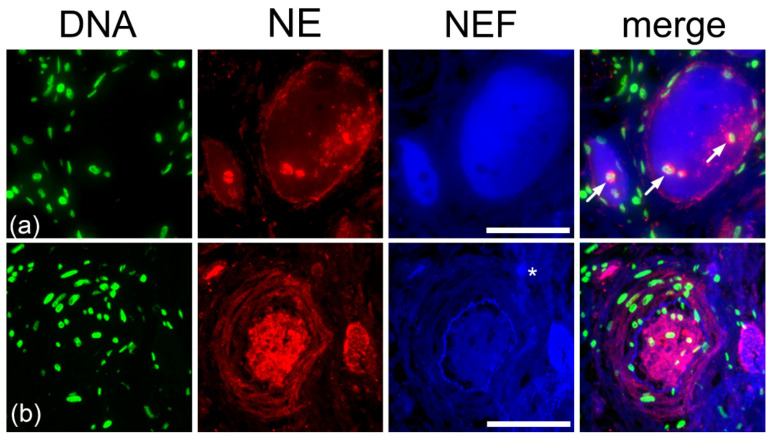
NE-rich and DNA-poor contents in vascular occlusion patients with COVID-19. (**a**,**b**) Varying composition of NE and DNA in label-free detected occlusion. Small vessels are marked with asterisks in the column showing NEF. Arrows indicate intravascular neutrophils with intact nuclei in columns showing merged images. Stains: DNA by propidium iodide (DNA, green), NE by fluorescence-labeled antibodies (NE, red) and native endogenous fluorescence (NEF, blue). The right column displays merged fluorescence. The scale bars represent 100 µm.

Stainings of NEF-detected occlusions with elastase and DNA showcase the second category of pleomorphic vascular occlusion (Figure 9). In this type, NE (I) spreads moderately across occlusions, (II) shows few neutrophils within the occluded structure, (III) encircles margins of the occlusion (Figure 9a), and (IV) a high amount clotts the vessel lumen and is to a great extent scattered throughout the vessel walls (Figure 9b). Propidium-iodide-stained cells show a fewer number of cells in contrast to DNA-rich NE-poor pleomorphism (Section 2.4 Type I pleomorphism). In addition, the lack of a NE-DNA barrier in Figure 9b suggests an impaired formation of NETs unable to protect the vessel wall from excessive injury. Occlusions from the consecutive area showed a low number of platelets in the clots and the absence of fibrinogen; the latter was observed only in the vicinity of the occluded vessel (Figure S8).The morphology of this category of type II occlusions is shown in H&E staining in the supplementary material Figure S10c,d.

### 2.6. Type III Pleomorphism: DNA- and NE-Rich

**Figure 10:**
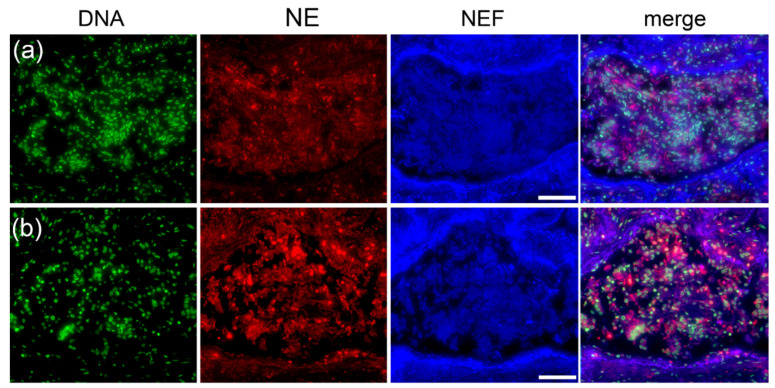
Large vessel with DNA- and NE-rich occlusions. (**a**,**b**) Co-staining for DNA and NE revealed areas of overlap signals of the two, exhibiting occurrence of NETs and some regions lacking NETs and only emitting NEF. Stains: DNA by propidium iodide (DNA, red), NE by fluorescence-labeled antibodies (NE, green), and native endogenous fluorescence (NEF, blue). The merged fluorescence is displayed in the right column (merge). The scale bar represents 100 µm.

In type III pleomorphism, vascular occlusions congesting large pulmonary vessels are rich in both DNA and NE. A large proportion of the occlusions showed co-localization for NE and DNA; however, some regions emit only high-intensity NEF signals (Figure 10).

Zones within the occlusions co-localizing for DNA and NE suggest a strong presence of NETs; however, this occlusion pleomorphism did not exhibit similar NETs forming barrier as observed in type I and type II pleomorphism and remained relatively centralized in the vessel lumen. These clots were absent for platelets and fibrinogen was found randomly scattered throughout the occlusions (Figure S9). The morphology of the type III occlusions is shown using H&E staining in the Supplementary Material Figure S10e,f.

### 2.7. Age and Gender Influence the Occurrence of Type of Pleomorphic Occlusion

Patient demographic and clinical characteristics are detailed in Table S2 in the supplementary material. As all patients admitted at the time of hospitalization were over 50 years of age, patient age was analyzed as one of the variables for influencing the type of pleomorphic occurrence. In addition, occlusions in the lung tissue specimen from all patients with COVID-19 were counted and categorized according to their pleomorphism in Table 1 alongside each patient’s demographic data.

**Figure 11:**
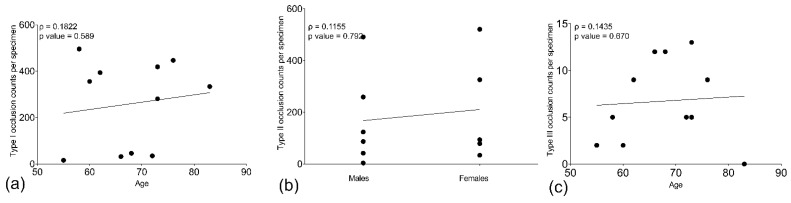
Spearman correlation for age and gender versus type I, type III, and type II pleomorphic occlusion occurrence in lung tissue specimens from patients with COVID-19, respectively. Spearman correlation coefficient (ρ) and *p* values for age with (**a**) type I pleomorphism of DNA-rich NE-poor occlusions, (**b**) males compared to females for type II NE-rich DNA-poor occlusions and (**c**) the age for type III DNA- and NE-rich occlusions showing a trend and small positive correlation.

Increased age was associated with a small but positive correlation between type I and III pleomorphic morphology, respectively (Figure 11a,c). In addition, females also showed a small but positive correlation for type II NE-rich DNA-poor occlusions compared to males (Figure 11b). Even though the *p* values of 0.589, 0.792, and 0.670 for type I, II, and III occlusions, respectively, remained not significant, the positive numbers from Spearman correlation coefficients obtained for age vs. type I pleomorphism (ρ = 0.1822), females vs. type II pleomorphism (ρ = 0.1155), and age vs. type III pleomorphism (ρ = 0.1435) showed a trend that age and female gender were directly related to type I, III, and type II occlusion morphology, respectively.

## 3. Discussion

We report a heterogenous presence of NE, DNA, platelets, and fibrinogen in vascular occlusions of patients with COVID-19. These occlusions can be detected at 488/525 nm wavelength primarily due to hemoglobin in the clots emitting native endogenous fluorescence (NEF). NE and DNA constituted significant components of the occlusions, imparting three types of pleomorphism. In addition to identifying pleomorphic occlusions using immunofluorescence staining, NEF reliably distinguished various shapes of clots, including small vessel occlusions and displayed arterial vessel walls. Therefore, NE and DNA showed morphological differences in the composition of immunothrombosis occluding pulmonary vessels to a large extent. Furthermore, these occluding immunothromboses can be detected by NEF without the necessity of extra H&E or immunofluorescence staining. Our results indicate that the observed occlusions comprised more-or-less healthy erythrocytes that released hemoglobin and its degradation products. These autofluorescent, endogenous compounds remain entrapped into the occlusion independent of their origin and cause robust NEF signals. Therefore, occlusions can be reliably detected by employing NEF as a label-free method.

Various endogenous fluorophores are known to contribute to NEF and, at particular wavelengths, can enable the detection of morphological changes in the vascular structure and indicate physiological abnormalities of blood cells. Several native fluorophores such as reduced NADPH, FAD, tryptophan, collagen, elastin, and hemoglobin in porphyrins are attributed to NEF [26,27]. We found that most occlusions detected at 488/525 nm were positive for hemoglobin and emitted NEF. A possible explanation could be the classification of the canonical thrombi as “red” or “white”. The former often form under low share flow in veins close to intact endothelium; they are rich in fibrin and encapsulate abundant red blood cells and activated platelets. The latter often occurs under high shear flow in arteries; they are rich in platelets and contain few red blood cells. Like all types of vascular clots, they tend to incorporate more or fewer NETs and various amounts of red blood cells. With time, these erythrocytes age, lose their membrane integrity, and release their hemoglobin into the vicinity of the cells. Interestingly, our results also show occlusion that is positive for NE and fibrinogen emitting NEF and several others that were negative for these antigens, including hemoglobin, and still emitting NEF. In addition, staining for platelets showed an absence of NEF emission. Overall, the emission of NEF is complex and an amalgamation of several entities from the vascular occlusion.

The immunofluorescently stained vascular occlusions exhibited several immunothrombi in the lungs of different patients with COVID-19 with a distinct morphology. These findings are, to some extent, in agreement with the two studies: firstly, Leppkes et al., 2020, reported dynamic morphology of vascular occlusions and Valdebenito et al., 2021, showed that SARS-CoV-2 induced lung damage is highly heterogeneous in terms of hemorrhagic events and immune cell infiltrations, with neutrophils as one of the crucial cell populations infiltrating the lungs [15,28]. In addition, our results reveal the presence of vascular occlusions mainly with heterogeneous deposits of NE and DNA in biopsies of patients with COVID-19. However, the precise mechanism underlying NE and DNA-mediated pleomorphic occlusions remains to be understood, and few recent studies have reported the varying constituents of clots in the vasculature of patients with COVID-19. Hemorrhagic events are associated with the destruction of blood vessels, resulting in a low influx of immune cells at the site of the collapse. As a result, many immune cells such as neutrophils, lymphocytes, and regular and spiky erythrocytes tend to form a border around the injury to contain lung damage [22]. These results exhibit occlusion with DNA-rich and NE-poor morphology sharing a similar pattern of cellular infiltration, specifically at the boundary of the clot where elastase and DNA overlapping signals indicate the formation of NETs preventing further lung injury.

Results from autopsies have mainly revealed exudative and proliferative organizing phases of diffuse alveolar damage (DAD), edema, capillary microthrombosis, neutrophilic capillarities, tracheobronchitis, multinucleation in type 2 pneumocytes, fibrosis, hyaline membrane, and alveolar hemorrhage. Reports from histological findings of patients’ biopsies with COVID-19 have revealed severe endothelial injury, pulmonary vessel and capillary thrombosis [29], and vessel occlusions in small to large arteries and veins [30,31] with the occurrence of thrombosis in about 60% of patients with COVID-19. These occluding thrombi extended from small to large sizes and obstructed the peripheral pulmonary arteries, both macroscopically and histologically [32].

The mechanism involving NETs has led to patients with severe COVID-19 being at increased risk of myocardial infarction due to the occlusion of cardiac vessels [33]. Furthermore, increased anti-SARS-CoV-2 IgA2 levels are a marker related to NET formation for more severe COVID-19 cases and poor outcomes [34]. Early reports of disease progression identified “cytokine storms” as pathognomonic in many patients. Many experienced pathological symptoms of acute respiratory distress syndrome (ARDS), with clinical reports showing neutrophilia and elevation in levels of several cytokines [35]. Additionally, atypical neutrophils with low buoyant density were found in patients with active disease and correlated to hypercoagulability [15,36,37]. These are reportedly prone to form NETs [38]. The high abundance of pro-inflammatory mediators and the “fragile” low-density neutrophils are considered to drive the thrombotic events found in patients with COVID-19 [36]. In addition to canonical thromboses, occlusion by NET aggregates of vessels has been reported [20].

The occurrence of pleomorphic immunothrombosis could be a result of two masters at work. One, the heterogeneous composition of immunothrombosis occluding pulmonary vessels in patients with COVID-19 might be intravascular NET formation, canonical thrombogenesis, or dysregulated immune system associated with an abnormal coagulation system. The building of immunothrombosis requires the cooperation of several pathways and cells, mainly neutrophils releasing NETs and NE granules, platelets clumping, and fibrinogen forming fibrin mesh structures. An impaired formation of immunothrombosis could comprise an insufficient or exaggerated involvement of any of these critical players, disabling the capture of invading SARS-CoV-2 intravascularly and causing further organ destruction [39].

Second is the influence of age and gender, given that both factors are associated with severe outcomes of COVID-19 disease [40]. Due to immobility, age is related to immune senescence and an inclination to a procoagulant state. Moreover, comorbidities contribute to an impaired immune system in the elderly [41]. As a result, the innate immune system is affected, and populations of dendritic cells, macrophages, and neutrophils decline [42,43,44]. In addition, the endothelial dysfunction with altered microcirculation, several coagulation factors, and platelet reactivity elevates [45], possibly leading to type I occlusions poor of neutrophil releasing NE but rich in cells from the hypercoagulation pathway.

On the other hand, studies report the formation of NETs in the elderly to be dysregulated. As a result, platelets are activated, promoting thrombogenesis and an exacerbated amount of NET formation. While the sera from patients with COVID-19 have been reported to have elevated amounts of NETs, mechanical ventilation for these patients can contribute significantly to the increase of NETs [22,46,47], which could explain the formation of type III pleomorphism in ageing patients where the occlusions are both DNA and NE rich.

Finally, the primary male hormone testosterone could lead to some tell-tale signs underlying type II pleomorphism. Low testosterone levels are a risk factor associated with fatal outcomes in males. In a study of 45 patients, most men who succumbed to death due to COVID-19 had low testosterone levels. In contrast, women with COVID-19 sent to the ICU had higher testosterone levels, indicative of hyperinflammation [48] and resulting in an increased influx of infiltrating neutrophils at the sites of SARS-CoV-2 vascular insult undergoing chromatin decondensation deposited with excessive amounts of NE granules forming NE-rich DNA-poor vessel occlusions more frequently in females than males. Interestingly, studies from COVID-19 non-survivors have been reported with heterogeneous conditions. While high neutrophil counts, lymphocytopenia, and low platelet counts were constant in recorded cases of non-surviving patients, fibrinogen levels fluctuated from low in some and elevated in others [49,50,51]. Most patients have mild thrombocytopenia, which is more pronounced in patients with a severe infection and a disease outcome that is not unusual in the case of COVID-19 [5,21]. Our results partially correlate with the findings previously reported. Neutrophilia can be associated with an exacerbated formation of NETs by low-density neutrophils providing a scaffold for thrombotic occlusions. In addition, low fibrinogen accompanied by high D-dimers and low platelets can suggest an ongoing disseminated intravascular coagulopathy and an increased thrombocyte turnover [2,21,52]. However, if not present in the occlusions or blood, the whereabouts and consumption of platelets and fibrinogen raise an intriguing question worthy of further investigation.

Our current study is not without limitations. Our study sample consists of only eleven patients who succumbed to COVID-19, so our correlation with age and gender is small but positive. An increase in the number of patients would perhaps show the influence of these variables affecting the occurrence of pleomorphism significantly. The analysis of various antigens in the occlusions via immunofluorescence staining requires a paraffin section having the same areas on tissues on several slides. Multi-sectioning of the paraffin tissue blocks creates more than 20 consecutive slides; the first and the last show somewhat different parts of the tissue. As a result, some tissue biopsies may appear slightly different from others. Therefore, we could not locate and evaluate exact occlusions for a few antigens.

## 4. Materials and Methods

### 4.1. Patients

The pathology institutes in Erlangen, Aachen, Regensburg, and Bayreuth provided anonymous tissue specimens. Specimens were obtained after all patients with the original strain of SARS-CoV-2 infection succumbed to death during the first wave of the pandemic in Germany. The extraction of some samples during autopsy depended on the site of worst macroscopic involvement or the lower right lung lobule in a central localization (close to the bronchi). A total of eleven patient specimens derived during autopsies post-death due to COVID-19 constituted the pleomorphism study cohort, with 6 males (54.5%) and 5 females (45.4%). The critical demographic characteristics of all 11 patients included in this study are presented in Section 2.7 of the results and in detail in the Supplementary Material (Table S2). We also compared the detection of occlusions using NEF from patients with COVID-19 to areas of non-occluded vessels in the lungs from 4 patients as the control group for patients with COVID-19 and 2 patients that died due to pulmonary embolism and acute cardiac failure. In addition, a popliteal artery aneurysm as a canonical occurrence of thrombosis (Figure S8) was also observed. The biopsies from patients enrolled in the control group as healthy donors were resected by forensic pathology after assessment of a non-diseased area of lungs. One patient showed signs of hyperemia/congestion and the remaining three patients from lung tumors.

### 4.2. Ethics Statement

All patients included in this study provided written and informed consent. All experiments were performed using human material according to the 1964 Helsinki Declaration and its later amendments or comparable ethical standards for research involving human subjects. In addition, institutional approval was obtained from each local Ethical Committee (permit #193_13B; permit # 174_20B; EK 092/20; EK 119/20; EK 460/20 approved on 07.05.2020, 02.04.2020, 24.06.2020, 07.12.2020, respectively).

### 4.3. Immunofluorescence Staining

Paraffinized lung tissue specimens were incubated for one hour at 94 °C for deparaffinization. Traces of any residual paraffin was removed by immersing the slides in ROTI Histol (Carl Roth Gmbh + Co. KG, Karlsruhe, Germany; Catalog # 6640.1) thrice for 8 min each. Lung specimens were rehydrated in decreasing alcohol concentrations (100%–96%–70%) (Carl Roth Gmbh + Co. KG, Karlsruhe, Germany, Catalog # 9866.6) for 5 min each. Epitopes were retrieved using a sodium citrate buffer at pH six at 90 °C (Merck KGaA, Darmstadt, Germany; Catalog # 106448) for 20 min. Specimen slides were cooled to room temperature for 30 min and blocked for 1 h with buffer containing 10% fetal calf serum (FCS) (ThermoFisher Scientific, Waltham, MA, USA; Catalog # 10270196), 2% bovine serum albumin (BSA) (ChemCruz, Dallas, TX, USA; Catalog # sc-2323A), 0.1% Triton X-100 (Sigma-Aldrich, St. Louis, MO, USA; Catalog # X100—1L), and 0.05% Tween 20 (Merck KGaA, Darmstadt, Germany; Catalog # 8.22184.100) in Dulbecco’s Phosphate buffer saline (1x DPBS) (ThermoFisher Scientific, Waltham, MA, USA; Catalog # 14190144). Specimen slides were then incubated with primary antibodies that stain for neutrophil elastase (NE) (1:100, Abcam, ab68672, RRID: AB_1658868), citrulline histone H3 (citH3) (1:300, Abcam, ab5103, RRID: AB_304752), platelet glycoprotein IIb or CD41 (1:100, Abcam, ab134131, RRID: AB_2732852), fibrinogen α chain (1:100, Abcam, ab92572, RRID: AB_10561758) and hemoglobin subunit α chain (1:300, Abcam, ab92492) in a humidified chamber at 4 °C overnight. In addition, the specificity of the elastase antibody was ensured and confirmed in non-occluded areas of the same tissue section. Tissue specimen slides were rinsed thrice with PBS. The slides were incubated with goat anti-rabbit secondary antibody conjugated with AlexaFluor647 (AF647) (1:400,# A32733, RRID: AB_2633282, ThermoFisher Scientific) and counterstained for DNA with propidium iodide (1:500, Sigma-Aldrich, St. Louis, MO, USA; Catalog # 4864) at room temperature for 1.5 h. The slides were rinsed twice with PBS and once with water. Negative controls were prepared simultaneously, lacking the primary antibody for each staining. The stained specimens were mounted in a DAKO fluorescence aqueous mounting medium (Agilent Technologies, Santa Clara, CA, USA; Catalog # S3023).

### 4.4. Hematoxylin and Eosin (H&E) Staining

Hematoxylin and eosin (H&E) staining is routinely performed in our histology laboratory. The specimens were deparaffinized for one hour at 94°C. Traces of any remaining paraffin were removed using ROTI-Histol (Carl Roth Gmbh + Co. KG, Karlsruhe, Germany; Catalog # 6640.1) twice for 5 min. Next, the sections were washed twice with 100% and 96% alcohol (Carl Roth Gmbh + Co. KG, Karlsruhe, Germany, Catalog # 9866.6) for 5 min at room temperature and distilled water. The staining procedure included incubation in Mayer’s hämalaun solution diluted in distilled water (Merck KGaA, Darmstadt, Germany; Catalog # 1.092490500) (1:10) for 10 min at room temperature. Slides were then washed with distilled water and Eosin Y solution (Sigma Aldrich, Merck KGaA, Darmstadt, Germany; Catalog # 318906-500 mL) for 15 to 45 min. Specimen slides were rinsed in distilled water, ascending alcohol series, and n-butyl acetate (Merck KGaA, Darmstadt, Germany; Catalog # 1.01974.2500). The specimens were mounted with ROTI mount synthetic mounting medium (Carl Roth Gmbh + Co. KG, Karlsruhe, Germany; Catalog # HP68.1).

### 4.5. CD31 Staining

Paraffin slides containing lung tissue specimens were incubated overnight at 60 °C for deparaffinization. Residual paraffin was removed by immersion in xylol twice for 15 min each. The tissue specimens were rehydrated using decreasing concentrations of ethanol (100%–96%–85%–70%) for 2 min each. Epitope retrieval was performed using target retrieval solution (TRS) at pH 9 at 95°C (Agilent Technologies, CA, USA; Catalog # S2367) for 20 min. The slides were cooled to room temperature for 20 min and rinsed with Tris buffer saline (1x TBS) twice for 5 min each. Endogenous peroxidases were blocked using 7.5% H_2_O_2_ in 70% ethanol for 10 min, and tissue specimens were rinsed with water thrice for 3 min each. Endogenous biotin was blocked using the avidin/biotin blocking kit (Vector Laboratories, Newark, CA, USA; Catalog # SP-2001). Subsequently, CD31 staining was performed using an anti-CD31 antibody (1:50, #M083, RRID: AB_2114471), followed by detection with RTU. Vectastain Kit with NovaRed as substrate (Vector Laboratories, CA, USA; Catalog # PK-7100) and counterstained using hematoxylin (Merck KGaA). The slides were mounted in a non-aqueous or permanent mounting medium (Vector Laboratories, CA, USA; Catalog # H-5000-60).

### 4.6. Native Endogenous Fluorescence (NEF)

**Figure 12:**
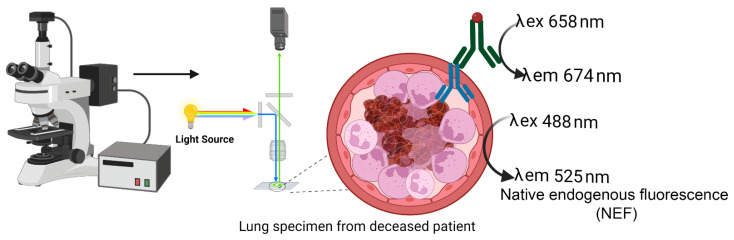
Schematic flow depicts label-free native endogenous fluorescence (NEF) acquisition coupled with indirect immunofluorescence staining. Paraffinized lung tissue specimens from Patients with COVID-19 were stained to detect neutrophil elastase (NE), a neutrophil extracellular trap formation marker, and visualized using a fluorescence microscope. The microscope equipped with red and blue laser enabled the excitation and emission spectra of Cy5 conjugated secondary antibody specific for primary antibody interacting to neutrophil elastase at 658/674 nm and NEF originating at 488/525 nm.

Fluorescence microscopes (Nikon ECLIPSE Ni; Keyence BZ-X700 E & Leica Versa 8) equipped with light sources enabled label-free native endogenous fluorescence (NEF) detection and immunofluorescence specific signals from neutrophil elastase, CD41 platelets, fibrinogen α chain, and hemoglobin α subunit. Targeted proteins of interest were recorded in the Cy5 channel at an emission wavelength of 674 nm and NEF at 525 nm shown in the schematic workflow in Figure 12.

### 4.7. Morphometry

We performed morphometry with Adobe Photoshop CC 2018 (San José, CA, USA) or Fiji Version 1.53k [53], employing self-developed modules.

### 4.8. Statistics

The areas between open and occluded vessels in lung tissue specimens from patients with COVID-19 and healthy controls were evaluated and compared using Fisher’s exact test. Statistical significance is indicated in the figures. Correlations of age and gender with types of pleomorphic occlusions occurring in lung specimens from patients with COVID-19 were evaluated by Spearman correlation two-tailed calculations. Statistical analysis and graphical visualizations were conducted using GraphPad Prism 8.3.0 software (GraphPad Software, San Diego, CA, USA) and Microsoft Excel 2019 (Microsoft, Bay Area, CA, USA).

## 5. Conclusions

Label-free NEF supported our histological evaluation of pleomorphic pulmonary occlusions. Our results show NE and DNA as the significant components imparting heterogeneity in the occlusions accompanied by the reduced amount or lack of platelets and fibrinogen. Therefore, we categorize our pleomorphic occlusions into three categories: DNA-rich NE-poor, NE-rich and DNA-poor, and DNA- and NE-rich. These findings are essential in better understanding the pulmonary pathology and structures of vascular occlusions, which contributes to understanding the composition of occlusions and improving anti-clotting strategies in patients with COVID-19 and other diseases characterized by immunothrombosis. Different forms of DNase and heparin are known to disintegrate NETs. A possible therapeutic intervention could be the utilization of DNases and heparin to dismantle vascular occlusion, whether DNA-rich NE-poor or NE-rich DNA-poor offering a disruption in the mesh-like chromatin web clotting the pulmonary vessels and imparting a protective intervention as an anti-clotting therapy for both elderly males and females. In addition, after assessment of hormonal levels upon hospitalization, testosterone or progesterone supplements could be provided to restore normal healthy hormone levels. However, these combinatorial therapeutic administrations as therapy require further investigation. In addition, future studies are necessary to explore which elements of the impaired immune system or the coagulation cascade cause morphological differences in the occlusions of patients with COVID-19.

## Figures and Tables

**Table 1:** Occlusions counts categorized morphology from each patient lung tissue specimen with corresponding anonymized demographic data.

				Pleomorphism in Occlusion		
Patient	Age (in Range)	Gender	Weight (in kgs)	Type I	Type II	Type III
1	58–62	Female	N/A	356	94	2
2	66–71	Female	100	46	34	12
3	71–75	Male	85	281	4	13
4	70–74	Female	120	35	79	5
5	81–85	Male	N/A	334	124	0
6	56–60	Male	N/A	496	259	5
7	74–78	Male	80	447	491	9
8	64–68	Male	97	32	87	12
9	71–75	Female	N/A	419	326	5
10	60–64	Female	N/A	394	521	9
11	53–57	Male	64	16	42	2

## Data Availability

The data that support the findings of our study are available from the corresponding author upon reasonable request.

## Supplementary Materials

The following supporting information can be downloaded at: https://www.mdpi.com/article/10.3390/ijms232315126/s1, Figure S1: Label-free detection using NEF detects a thrombus occlusion; Figure S2: NEF accurately detects occlusion of large and microvessel pulmonary occlusions; Figure S3: A large partially occluded vessel surrounded by several clots were detected by NEF and shown in H&E; Figure S4: Non-occluded vessels in normal control lungs were detected using the label-free NEF; Figure S5: NEF shows several non-occluded alveolar septa in control normal lungs; Figure S6: Neutrophil elastase (NE)-rich arterial vessel wall have either low or lack of platelets and fibrinogen; Figure S7: DNA-rich and NE-poor occlusions contain lower amounts of platelets (CD41 positive) and fibrinogen; Figure S8: NE-rich and DNA-poor occlusion areas are low for platelets and lack fibrinogen; Figure S9: DNA and NE-rich occlusions harbor deposition of fibrinogen but lack platelets; Figure S10 H&E stained lung specimen shows the original morphology of three types pleomorphic occlusions in patients with COVID-19; Table S1: Pathology reports from professional pathologists who evaluated the lung tissue specimen based on H&E staining and CD31 immunohistochemistry; Table S2: Demographic table with clinical data recorded from patients with COVID-19 enrolled in the study cohort. Figure S1: title; Table S1: title; Video S1: title.
