# Supplementary material for: A Pleomorphic Puzzle: Heterogeneous Pulmonary Vascular Occlusions in Patients with COVID-19
Source: Int J Mol Sci. 2022 Dec 1;23(23):15126. doi: 10.3390/ijms232315126 (PMC9739020; doi:10.3390/ijms232315126)
Supplement: Supplementary file 1 [file ijms-23-15126-s001.zip › ijms-1951392-supplementary.pdf]

**Supplementary Material**

**Reagent validation**

There were no animal models or cell lines used in this study. Our lab previously validated primary and secondary antibodies for neutrophil elastase (NE) in human tissues and utilized them for immunofluorescence staining. Specificity and optimal dilutions for platelet glycoprotein IIb or CD41, fibrinogen $\alpha$-chain and hemoglobin-$\alpha$ subunit for immunofluorescence staining were determined using paraffinized lung tissue specimen slides from human autopsy material and in the presence of appropriate controls

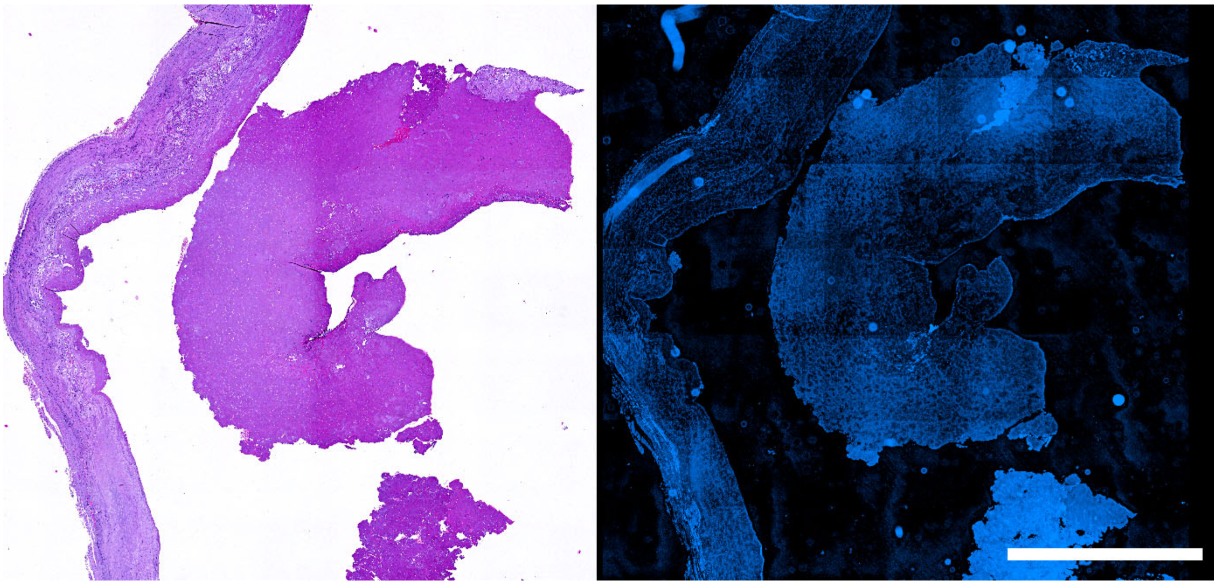

**Figure S1** Label-free detection using NEF detects a thrombus occlusion. (Left) Hematoxylin & Eosin (H&E) stained shows a canonical thrombus occluding a vessel in a tissue specimen from a patient with a popliteal artery aneurysm (Right). NEF (in blue) shows the detection of the same occlusion. The scale bar represents 1000 μm

**H&E vs NEF**

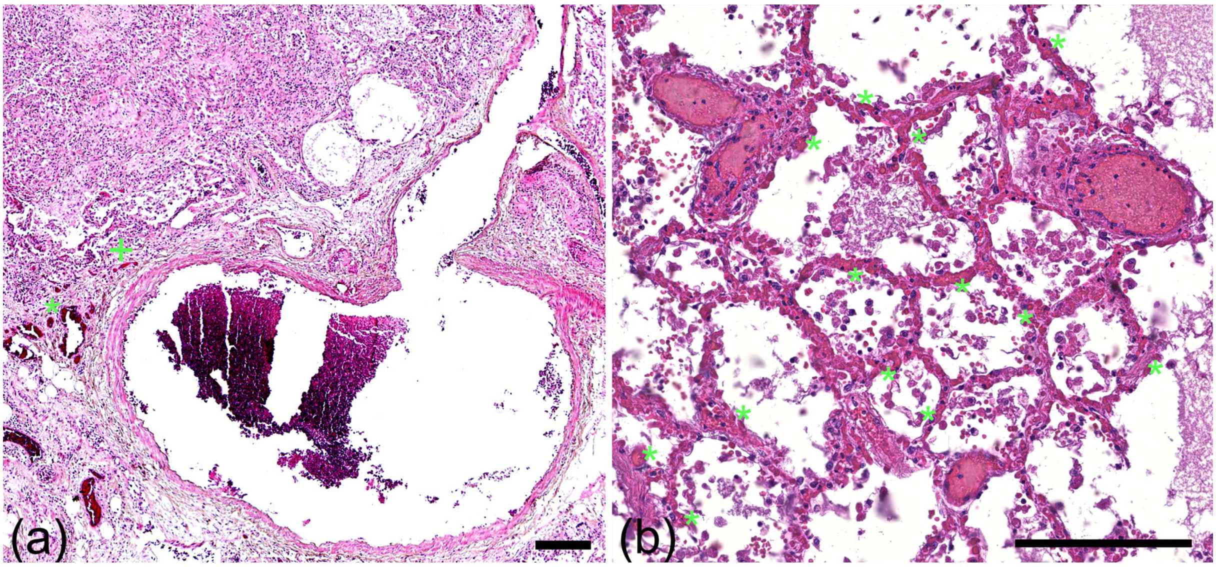


**Figure S2.** NEF accurately detects occlusion of large and microvessel pulmonary occlusions. A representative image of a NEF identified pulmonary occlusions in figures 1, and figure 2 of the main manuscript are shown above in H&E staining which compares and confirms the NEF coupled immunofluorescence image, illustrating the specificity of NEF to recognize vessel shape heterogeneity. The H&E image shows longitudinal cross-sections (+), circular vessels (*) of different sizes (a) and microvessel clots in the alveolar region (b). The scale bars represent 600 μm in (a) and 250 μm in (b).

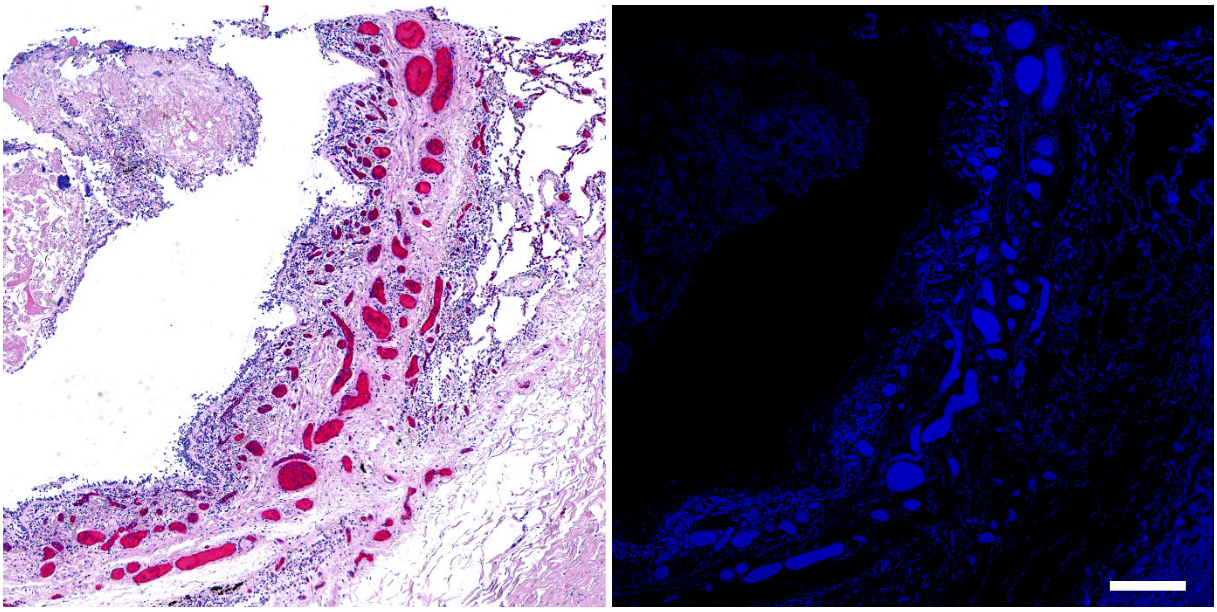

**Figure S3** A large partially occluded vessel surrounded by several clots were detected by NEF and shown in H&E. A representative H&E stained image of several small occlusions surrounding a partly clotted large pulmonary vessel (left) confirms the accuracy of the same vessel's fluorescence image at 488/525 nm (right, blue). The scale bars represent 100 μm

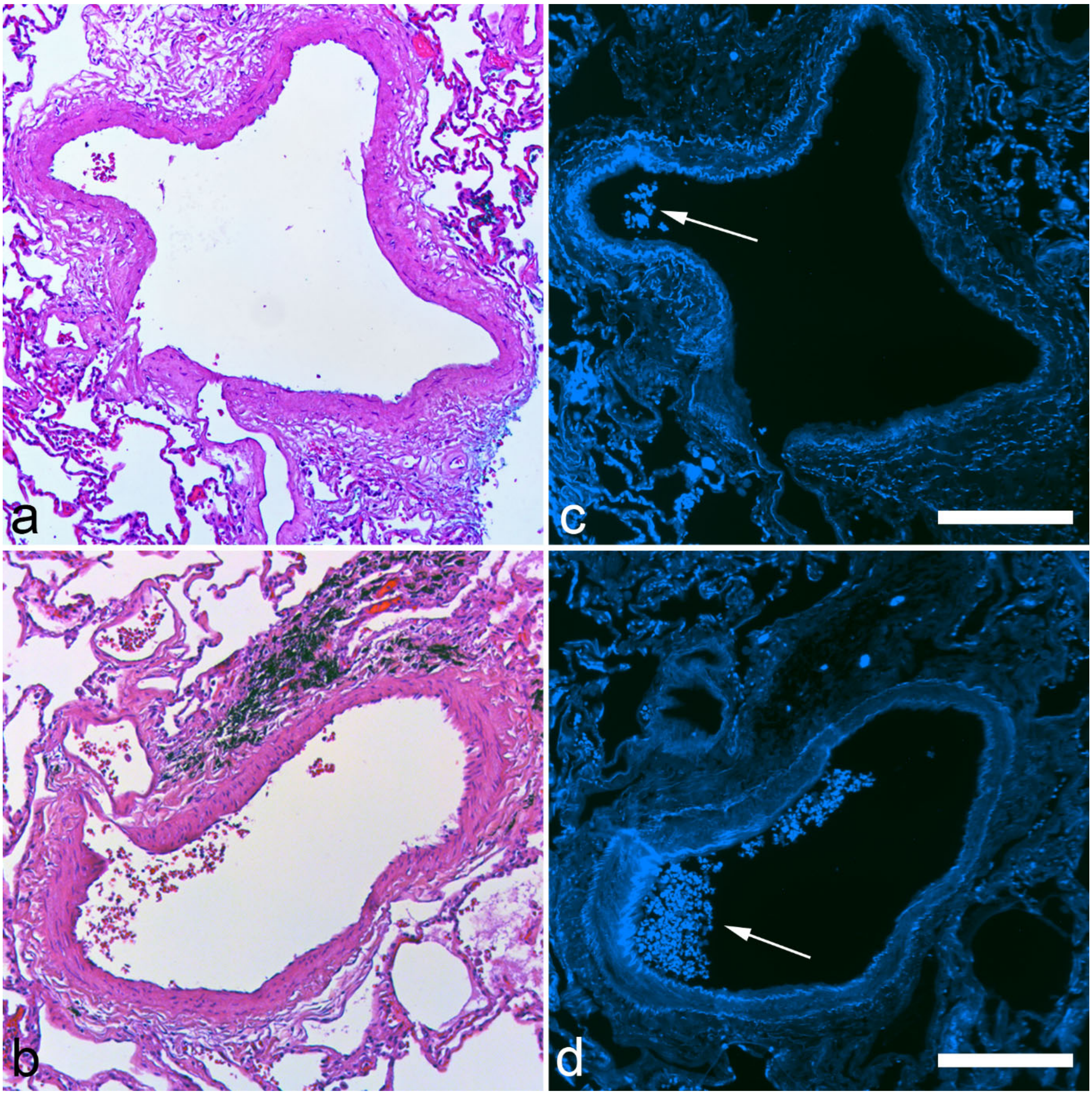


**Figure S4** Non-occluded vessels in normal control lungs were detected using the label-free NEF. H&E stained (a,b) and NEF signals in blue (c,d) show two open pulmonary vessels with surrounding vascular structure and the presence of a few erythrocytes (white arrows) within the vessel lumen. The scale bars represent 250 μm.

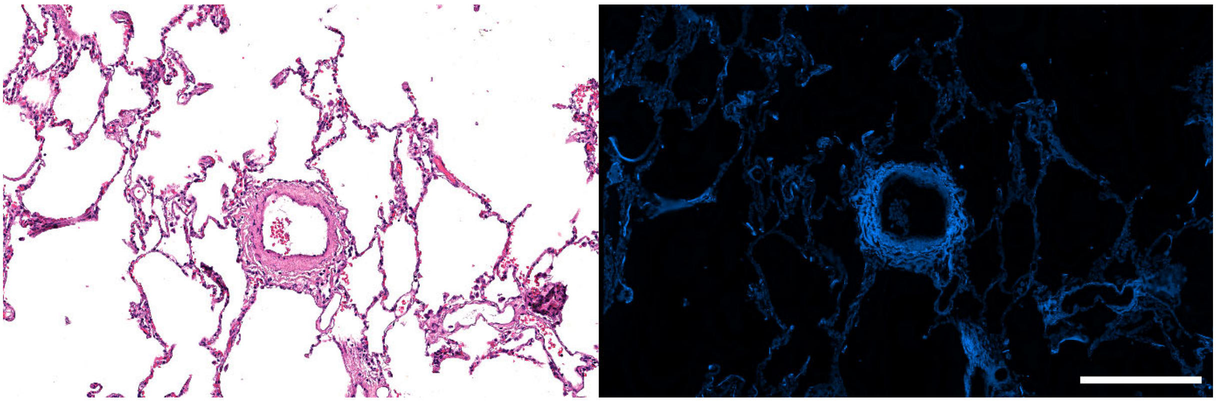

**Figure S5** NEF shows several non-occluded alveolar septa in control normal lungs. (Left) H&E staining of a typical control healthy lung. (Right) NEF signals (blue) of the same alveoli network surrounding a vessel. As shown, H&E staining and NEF identified the alveoli region in normal control lungs. The scale bar represents 250 μm.

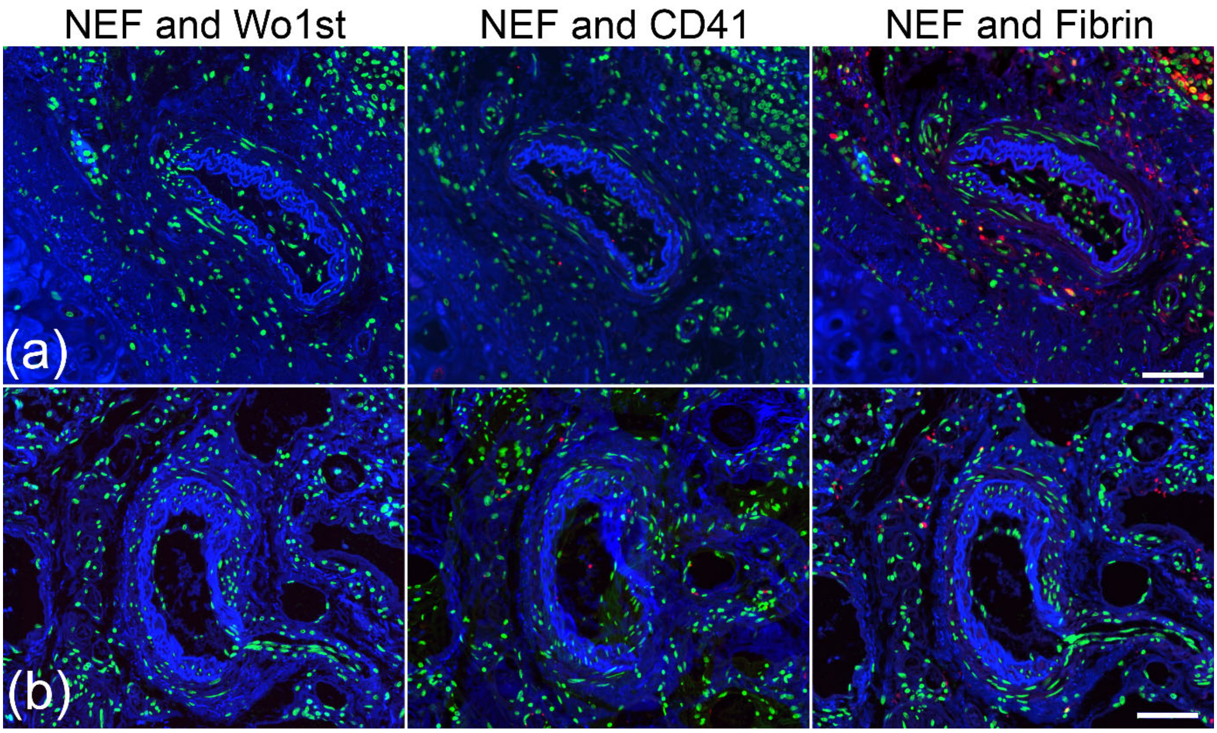


**Figure S6** Neutrophil elastase (NE)-rich arterial vessel wall have either low or lack of platelets and fibrinogen. (a, b) NEF detected arterial walls, as shown in figure 8 of the main manuscript, show either a reduced or a lack of platelets (CD41 positive, red) fibrinogen (red) in the vessel walls. DNA was stained with propidium iodide (green). The scale bars represent 200 μm.

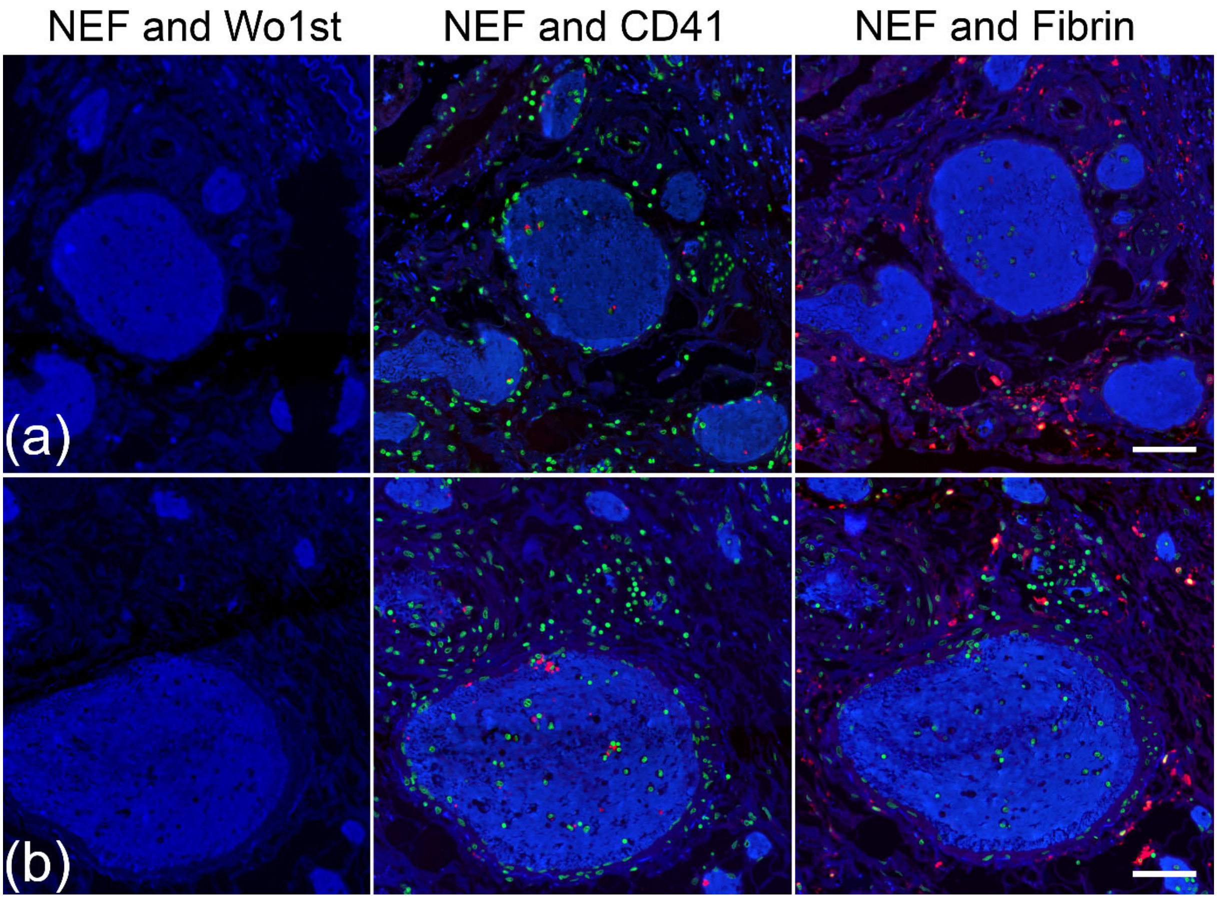


**Figure. S7** DNA-rich and NE-poor occlusions contain lower amounts of platelets (CD41 positive) and fibrinogen. (a, b) Immunothrombosis occluding pulmonary vessels with DNA-rich and NE-poor contents, as shown in figure 8 of the main manuscript, have reduced number of platelets (CD41 positive, red) and fibrinogen (red) shown above in a and b. DNA was stained with propidium iodide (green). The scale bars represent 200 µm.

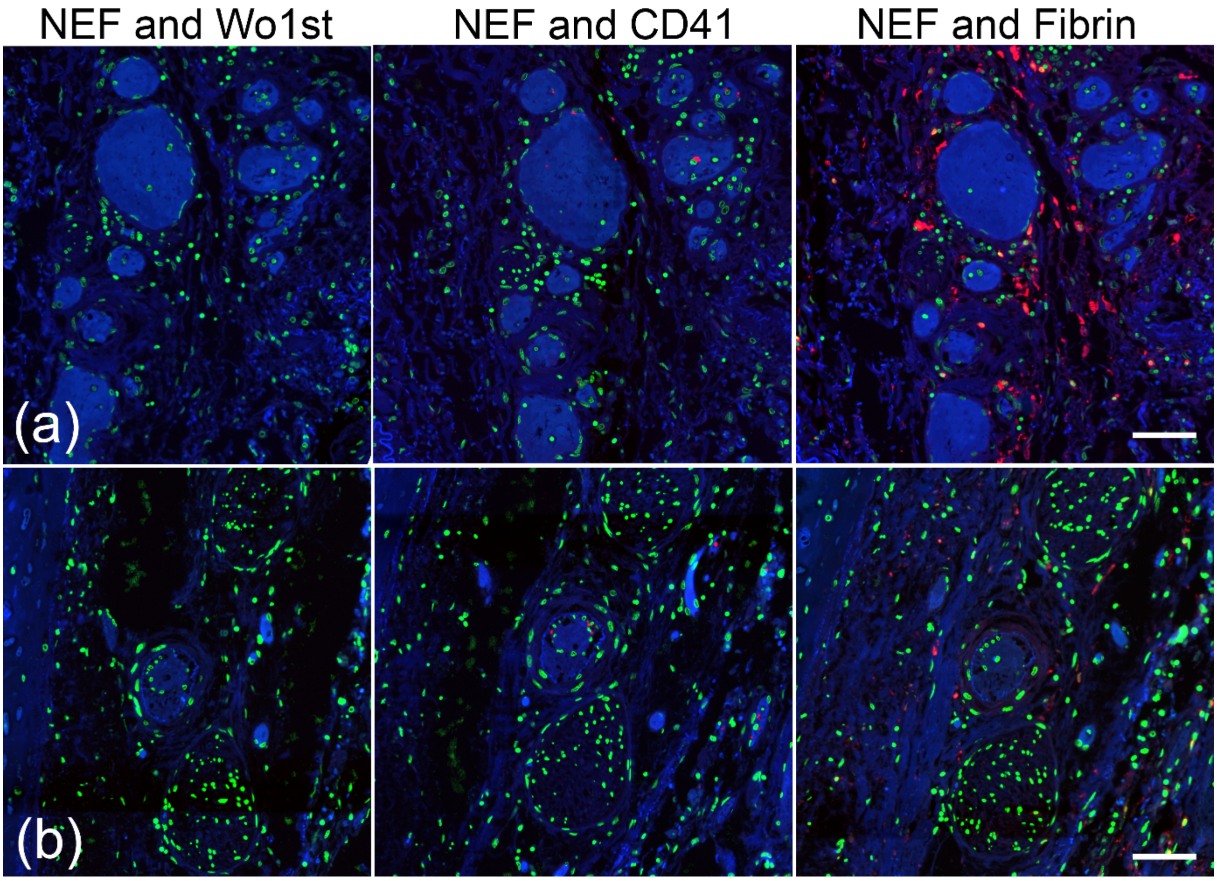


**Figure. S8** NE-rich and DNA-poor occlusion areas are low for platelets and lack fibrinogen. (a, b) Occlusions with type II pleomorphism as shown in figure 9 of the main manuscript show less platelets (CD41 positive, red) and lack fibrinogen (red), with fibrinogen present only around the occlusions (a). DNA was stained with propidium iodide (green). The scale bars represent 200 μm.

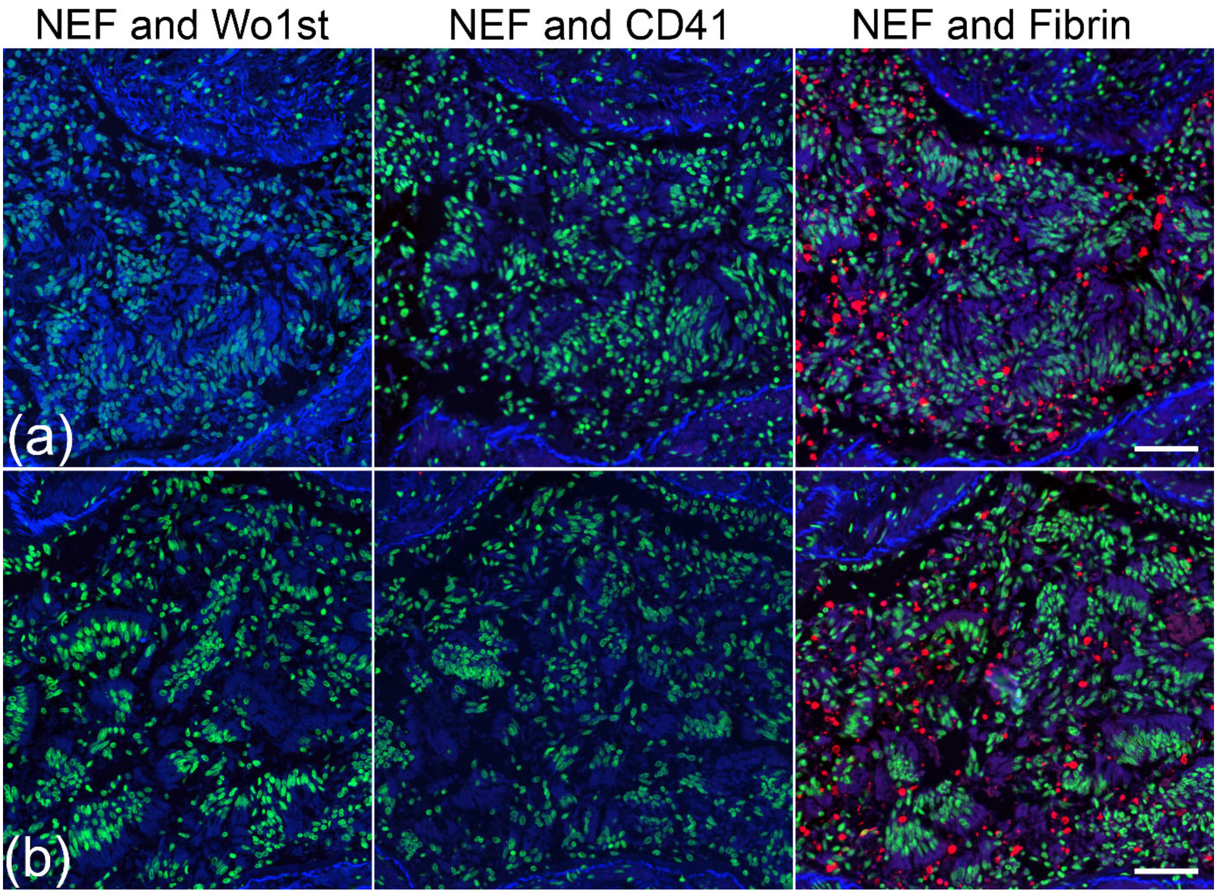


**Figure S9** DNA and NE-rich occlusions harbor deposition of fibrinogen but lack platelets. (a, b) Large pulmonary vessel occluded as shown in figure 10 of the main manuscript show lack of platelets (CD41 positive, red) but showed a randomly scattered presence of fibrinogen (red) throughout the occluding immunothrombus. DNA was stained with propidium iodide (green). The scale bars represent 200 μm.

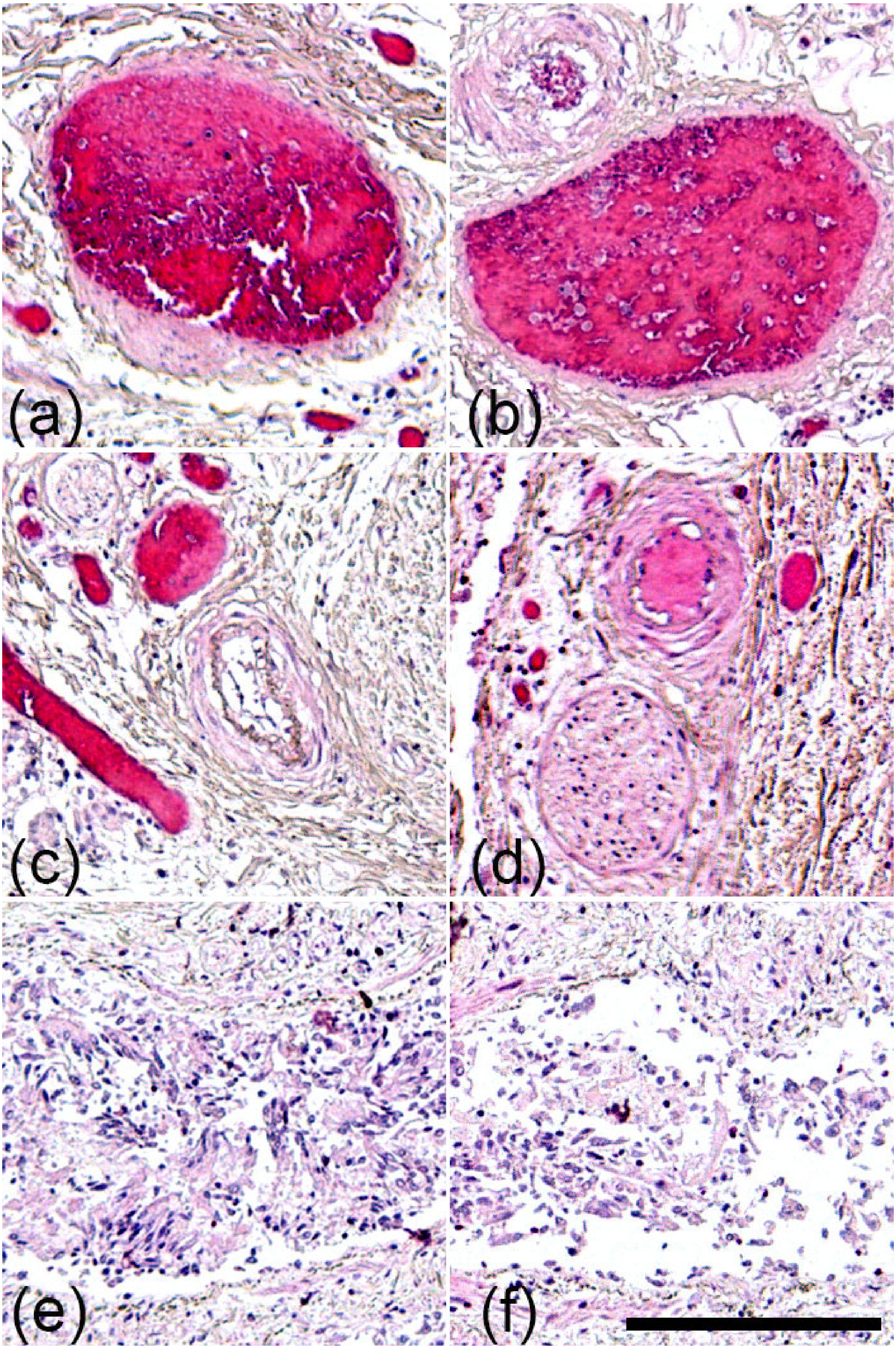

**Figure S10** H&E stained lung specimen shows the original morphology of three types

pleomorphic occlusions in patients with COVID-19. Composite exhibits the morphology of three types of pulmonary pleomorphic occlusions observed in patient biopsies (a,b) type I pleomorphism of DNA-rich and NE-poor (c,d) type II pleomorphism of NE-rich and DNA-poor and (e,f) type III DNA-and NE-rich pleomorphism. Scale bar represents 400 μm.

**Table S1:** Pathology reports from professional pathologists who evaluated the lung tissue specimen based on H&E staining and CD31 immunohistochemistry. These specimen were derived during the autopsies of patients who succumbed to COVID-19 infection and enrolled in this study's cohort.

| Patient. | Pathology reports |
|---|---|
| 1 (CD31 staining) | Pulmonary edema with fluid and some inflammatory cells in most of the alveolae. Thickened and hypervascularized alveolar septae with already some hyaline membranes (ARDS). One pulmonary vein with fresh non-adherent thrombus. |
| 2 (H&E) | Signs of septic circulatory failure: Severe, florid lateral abscessing, focally also already in the organization, subpleural panlobular bronchopneumonia; in the upper and lower lobe of the right lung.Angioinvasive mycosis in the right upper and lower lung lobes (subtyping in progress) acute blood stasis of the lungs; pronounced pulmonary edema; single small thrombocytes small, partially organized thrombi in small peripheral pulmonary arteries in peripheral pulmonary arteries in the upper and lower lobes of the left lung. |
| 3 (H&E) | Small-cell angioinvasive mycosis in the area of the right upper lobe of the lung; marked hemorrhages into the into the lung parenchyma; accompanying fibrinous pleurisy; acute blood congestion of the lungs. |
| 4 (H&E) | Diffuse alveolar damage (DAD), proliferative/organizing phase, edema, hyaline membranes, squamous metaplasia, multinucleated giant cells, breast cancer pulmonary metastases. |
| 5 (H&E) | DAD, proliferative/organizing phase with bacterial superinfection, edema, hyaline membranes. |
| 6 (H&E) | DAD, fibrotic phase, squamous metaplasia, multinucleated giant cells. |

| | |
|---|---|
| 7 (H&E) | DAD, proliferative/organizing phase, edema, hyaline membranes, emphysema. |
| 8 (H&E) | DAD, proliferative/organizing phase with bacterial superinfection and hemophagocytic lymphohistiocytosis, edema, hyaline membranes, squamous metaplasia, multinucleated giant cells. |
| 9 (H&E) | DAD, proliferative/organizing phase, tracheobronchitis,pulmonary microthrombi,Edema,squamous metaplasia, multinucleated giant cells. |
| 10 (H&E) | DAD, exudative phase, intraalveolar edema, hemosiderin-laden macrophages, papillary thyroid cancer pulmonary metastases. |
| 11 (H&E) | Diffuse alveolar damage, proliferative/organizing phase with bacterial superinfection, tracheobronchitis, pulmonary artery thrombosis. |

**Table S2.** Demographic table with clinical data recorded from patients with COVID-19 enrolled in the study cohort

| **Patient.** | **Age** | **Gender** | **Weight (in Kgs)** | **Body Mass Index (BMI)** | **Comorbidities** | **Treatment** | **WBC counts before death** | **Lymphocyte counts before death** | **Platelet counts before death** | **COVID-19 antibodies *** |
|---|---|---|---|---|---|---|---|---|---|---|
| 1 | 58-62 | Female | N.A | N.A | Depression | N.A | 16,9 /nl | 1,5 /nl | 58 /nl | N.A |
| 2 | 66-71 | Female | 100 | 35,4 | Hypertension, obesity and depression | steroids (vasoplegia) | N.A | N.A | N.A | N.A |
| 3 | 71-75 | Male | 85 | 26,2 | Hypertension, COPD, obesity, HLP and NASH | steroids (vasoplegia) | N.A | N.A | N.A | N.A |
| 4 | 70-74 | Female | 120 | 27 | Ischemic cardiomyopathy, myocardial infarction, breast cancer, liver cirrhosis, lymphocytic thyroiditis | ASS, Heparin | $12*10^3/\mu L$ | $2,7*10^3/\mu L$ | $177*10^3/\mu L$ | N.A |
| 5 | 81-85 | Male | N.A | 27 | Diabetes mellitus type II, cardiac insufficiency, ischemic cardiomyopathy, coronary artery disease, peripheral vascular disease, carotid vascular disease, previous smoker, hypercholesterinemia, arterial hypertension | N.A | $5,3*10^3/\mu L$ | $11,4*10^3/\mu L$ | $135*10^3/\mu L$ | N.A |
| 6 | 56-60 | Male | N.A | 25 | Heart valve prosthesis, arterial hypertension, diabetes mellitus type II, adipositas grade I, hyperlipoproteinemia | N.A | 25,3/nl | N.A | 61/nl | N.A |
| 7 | 74-78 | Male | 80 | 32 | Diabetes mellitus type II, dementia, ulcerative colitis | N.A | 9,3/nl | N.A | 315/nl | N.A |
| 8 | 64-68 | Male | 97 | 29 | Aortic aneurysm, adipositas, arterial hypertension, hypertensive cardiomyophathy | N.A | 10,2/nl | 6% | 83/nl | N.A |
| 9 | 71-75 | Female | N.A | 27 | Cardiac insufficiency, osteoporosis | Heparin, Argatroban, Heparin | 17,6/nl | 0% | 107/nl | N.A |
| 10 | 60-64 | Female | N.A | 24 | Atherosclerosis, kidney transplant, myocardial infarction, coronary atherosclerosis, thyroid cancer | N.A | 23/nl | 20% | 175/nl | N.A |
| 11 | 53-57 | Male | 64 | 23 | N.A | N.A | 9,5/nl | 3% | 48/nl | N.A |

*Measurement of COVID-19 antibodies did not exist in the first months of the pandemic; therefore, these values were not documented
